# Analysis on suicide risk in medical students, an analysis based on socio-formative conceptual mapping

**DOI:** 10.3389/fpubh.2026.1821748

**Published:** 2026-04-29

**Authors:** María Elena Calles-Santoyo, Elizabeth Reyna-Beltrán, Gerardo García-Maldonado, José Eugenio Guerra-Cárdenas

**Affiliations:** Faculty of Medicine of Tampico “Dr. Alberto Romo Caballero”, Autonomous University of Tamaulipas, Tampico, Tamaulipas, Mexico

**Keywords:** conceptual mapping, medical students, psychological predictors, risk factors, suicide risk

## Abstract

This article reports a documentary research project using a conceptual mapping methodology for suicide risk in medical students, emphasizing a socio-formative approach. The purpose is to define suicide risk and, through a socio-formative approach, identify and reduce suicide risk in medical students by understanding risk factors and psychological predictors. The axes of the conceptual mapping are constructed through the analysis of scientific literature indexed in high-impact databases (Scopus, Web of Science, PubMed, and PsycINFO) published between 2015 and 2025. The results highlight the complexity and multifactorial nature of suicide risk in this vulnerable population, emphasizing the interconnectedness of individual psychological, academic, psychosocial, and institutional factors. The conceptual mapping offers a comprehensive view of the research landscape, facilitating the identification of key areas for intervention, prevention, and future research.

## 1 Introduction

The well-being and mental health of medical students have emerged as a significant concern in academic research and educational institutions globally. The demanding nature of medical training, characterized by a heavy academic workload, constant stress, competitiveness, and early exposure to human suffering, predisposes this population to a higher risk of developing mental health problems, including depression, anxiety, and, in extreme cases, suicidal ideation and behavior (1, 2). These factors need to be addressed within the family by promoting aspiring medical students with solid mental and emotional health. However, this is not always the case, which causes various problems that accumulate throughout the student’s academic career and must be addressed by the institution’s academic staff.

The pedagogical approach of socioformation seeks to articulate meaningful learning regulated by metacognition (3). It also promotes the integration of theoretical and practical knowledge with an ethical and human dimension, which is key in clinical practice (4), and enhances the development of cross-cutting skills, such as emotional self-regulation, empathy, teamwork, and resilience, elements that directly impact students’ academic performance and mental health (5).

Suicidal ideation and suicide attempts among medical students not only represent an individual tragedy but also have significant implications for the future of the medical profession and the quality of patient care. Doctors in training with mental health issues may experience a decline in their academic and professional performance, as well as an increased risk of burnout and medical errors (6). Recent systematic reviews confirm the alarming prevalence of these problems. A meta-analysis reported that medical students experience higher rates of suicide and suicidal ideation compared to the general population, with significant stressors including academic pressure, challenges in personal relationships, and professional expectations (7). Another systematic study reports wide ranges of prevalence for conditions such as anxiety (7.04–88.30%), burnout (7.0–86.0%), depression (11.0–66.5%), stress (29.6–49.9%), and suicidal ideation (3.0–53.9%).

In this context, the persistence of these high rates and the breadth of the ranges reported in multiple systematic reviews within the 2015–2025 period underscore the severity and persistent nature of the problem. This suggests that educational settings have not developed sufficient strategies or have not effectively addressed the root cause, pointing to a mental health crisis that requires urgent reassessment and a more proactive and systemic approach on the part of educational institutions (8, 9). The pedagogical approach of socio-formation seeks to facilitate meaningful learning guided by metacognition (3), it also promotes the integration of theoretical and practical knowledge with an ethical and human dimension, which is key in clinical practice (4), and fosters the development of cross-cutting competencies, such as emotional self-regulation, empathy, teamwork, and resilience—elements that directly impact students’ academic performance and mental health (5). Socio-formation offers strategies aimed at developing individuals on personal, social, and professional levels, enabling them to integrate into society with strong ethical values, while promoting the individual’s holistic development. It represents a continuous process that not only generates knowledge but also contributes to cognitive, emotional, and social maturation (10). Within this approach, socio-formation views co-evaluation, self-evaluation, and peer evaluation as key tools in a meaningful assessment process aimed at demonstrating learning outcomes (11, 12).

Although suicide risk has been extensively studied from quantitative perspectives—through epidemiological research, psychometric scales, and statistical analysis—it is essential to explore its more complex and contextual dimension through qualitative methodologies. Therefore, the objectives of this research are: (1) to conceptualize suicide risk from a socio-formative perspective, (2) to identify risk factors and psychological predictors of suicide risk, and (3) to describe a proposal that contributes to identifying risk factors and psychological predictors and provides solutions from a socio-formative perspective.

## 2 Materials and methods

### 2.1 Study type and design

A qualitative document review was conducted using a design that employs the eight axes of conceptual mapping: Notion, Categorization, Characterization, Differentiation, Classification, Linkage, Methodology, and Exemplification (10). According to the author of this framework, these are key elements in which a construct or phenomenon to be studied can be understood and analyzed to give rise to new knowledge, in addition to explaining how a specific phenomenon relates to other theoretical concepts (constructs) or observable realities, from two approaches: logical and factual (13).

### 2.2 Guiding questions

Table 1 shows a key question for the development of the research work for each axis of the conceptual mapping.

**Table 1:** Conceptual mapping axes.

Central axis	Guiding question
Notion	How has the concept of suicide risk been addressed in scientific literature?
Categorization	What dimensions recur in the literature on this topic (psychological, emotional, contextual)?
Characterization	What are the main features of suicide risk that can be observed in a student?
Differentiation	How does suicide risk differ from other constructs?
Classification	How is suicide risk classified?
Linking	What logical and factual connections exist between suicide risk and constructs such as hopelessness, anxiety, and burnout in medical students?
Methodology	How has suicide risk been addressed in the academic environment to identify, prevent, and intervene in this condition?
Exemplification	What situations can contribute to evidence of suicide risk in medical students?

This table shows the structure of the conceptual mapping axes on which the review is based.

### 2.3 Inclusion criteria

Articles that met the following criteria were included:

- Peer-reviewed articles.
- Original studies, systematic reviews, meta-analyses, and qualitative studies with a clinical or educational focus.
- Published between January 2015 and May 2025.
- Languages: English and Spanish.
- Target population: undergraduate medical students.

### 2.4 Data source

To prepare this literature review, an exhaustive search of scientific literature was conducted in high-impact databases recognized for their relevance in the fields of medicine, psychology, and the social sciences. The selected sources were: PubMed, Scopus, Web of Science, and PsycINFO.

### 2.5 Search strategy

Key search terms and their Boolean combinations, tailored to each database, were used. The search terms were developed through an iterative process involving multiple stages to ensure the comprehensiveness and relevance of the strategy. First, we began by identifying the core concepts of the phenomenon under study: medical students, suicide risk, and associated psychological risk factors and predictors. Based on these thematic areas, we consulted the thesauri and controlled vocabularies of the selected databases, reviewing the MeSH (Medical Subject Headings) terms for PubMed, Emtree for Scopus, as well as the specific thesauri of PsycINFO and Web of Science. This review allowed us to identify the most appropriate standardized terms, such as “students, medical,” “suicidal ideation,” “suicide,” “risk factors,” and “depression.”

Subsequently, an initial review of previous systematic reviews in the field of mental health among medical students was conducted, with the aim of identifying terms commonly used in the specialized literature and ensuring the inclusion of keywords that have proven to be relevant in previous studies. Once an initial set of terms was defined, pilot searches were conducted using different Boolean combinations (AND, OR operators) and terminological variations to assess the sensitivity and specificity of the results. Finally, each search strategy was adapted to the specific syntax of each database—PubMed, Scopus, Web of Science, and PsycINFO—taking into account search field filters (title, abstract, keywords) and the established publication year limits (2015–2025). Some of the terms used included: “medical students,” “suicidal ideation,” “suicide risk,” “mental health,” “depression,” “anxiety,” “hopelessness,” “alexithymia,” “perfectionism,” “stress,” “burnout,” “psychological factors,” “risk factors,” and “predictors.”

### 2.6 Data selection and extraction process

The selection of articles was conducted in two phases. In the first phase, the records obtained from database searches were imported and managed in EndNote, software that allowed for the organization of references and the removal of duplicates. Subsequently, the relevance of the articles was assessed based on their titles and abstracts, applying the inclusion criteria. In the second phase, the preselected articles were read in their entirety to confirm their eligibility and extract relevant information on risk factors and psychological predictors of suicide risk. During this phase, EndNote was used for reference management and Microsoft Excel for documenting and organizing the extracted data, facilitating the monitoring of the selection process and the synthesis of the information.

### 2.7 Analysis and synthesis strategy

For a study based on the socioformative approach—which focuses on solving contextual problems, complex thinking, metacognition, and the collaborative construction of knowledge—the information coding process that best aligns with its principles is the three-stage process derived from grounded theory: open coding, axial coding, and selective coding. Open coding, originating in grounded theory, consists of examining the data inductively, ensuring that the analysis is guided by emerging content rather than by pre-established ideas or categories. In axial coding, the researcher connects the open codes to group them into broader categories that integrate a higher-order meaning. Finally, selective coding is the final stage of grounded theory coding, where the researcher integrates and validates the categories and relationships from the axial coding stage. The researcher focuses on the central category and relates it to the other categories, forming a central narrative or theory (14, 15).

## 3 Results

Although this study does not strictly adhere to the guidelines for a systematic or scoping review, a systematic search and selection process was followed to ensure the transparency and reproducibility of the findings. The initial search in the Scopus, Web of Science, PubMed, and PsycINFO databases yielded a total of 1,247 records (PubMed: 438, Scopus: 352, Web of Science: 289, PsycINFO: 168). After removing duplicates using EndNote, 892 unique records were obtained.

During the selection phase based on title and abstract, applying the predefined inclusion criteria, 765 records were excluded for not meeting the target population criteria or for not addressing the phenomenon of interest. A total of 127 articles were preselected for full-text evaluation.

Of these, 60 articles were excluded during the full-text review for the following reasons: failure to disaggregate data specific to medical students, failure to report risk factors or psychological predictors, being previously unidentified duplicate studies, or not being available in full text. Finally, 67 articles met all inclusion criteria and were subjected to qualitative analysis. They were predominantly cross-sectional studies, primarily originating from the United States, Latin America, China, and the United Kingdom, and most included students from different academic years.

### 3.1 Notion

Suicide risk is a construct whose main evidence is that the individual has suicidal ideation, plans, or has attempted suicide. This construct is complex and dynamic in nature, transcending the simple presence of thoughts of death to encompass interrelated emotional, cognitive, contextual, and behavioral dimensions (16, 17). Koppmann (18) addresses suicide risk as the interaction of personal, family, and social factors that lead the individual to attempt to take their own life. Therefore, suicide represents a serious mental health problem, considered the third leading cause of death worldwide (19). “It is an alarming public health problem, as it brings with it endless personal, social, and economic consequences” (20).

A critical component in suicide risk prevention is timely care. Epidemiological studies indicate that between 50 and 80% of suicide attempts have a history of previous attempts (21). From a socio-educational perspective, suicide risk is addressed by activating prior knowledge to develop psychosocial skills that enable students to resiliently face the challenges of their life journey. This perspective seeks to prepare them to resiliently face the challenges inherent in their life development. This model is based on principles aligned with the demands of the globalized world, such as active participation, social commitment, collaborative integration, and the appropriate use of technology (22).

### 3.2 Categorization

Suicidal risk can be categorized into at least three fundamental dimensions: psychological, academic, and social, which, when interacting, create a psychoeducational environment prone to emotional deterioration (5).

*Individual psychological factors*: This category emerges as a central element in predicting suicidal risk in medical students. Depression and anxiety are highly prevalent conditions in this population, significantly associated with suicidal ideation (1, 23). Prevalence rates for depression range from 11.0 to 66.5%, and for anxiety from 7.04 to 88.30%. Current scientific evidence identifies depression and emotional dysregulation as key predictive factors in assessing suicidal risk. These components, characterized by their impact on adaptive capacity and cognitive-emotional processing, require a comprehensive approach that combines early detection with socio-formative intervention strategies aimed at developing psycho-affective competencies and self-regulation mechanisms. Hopelessness, defined as the lack of positive expectations about the future, has been consistently identified as a powerful predictor (24). A multivariate analysis revealed that severe hopelessness significantly increased the likelihood of suicidal behavior.

Alexithymia, or difficulty identifying and expressing emotions, can hinder help-seeking and increase vulnerability to stress and suicidal risk (25). Dysfunctional perfectionism, characterized by excessively high standards and severe self-evaluation, can generate intense pressure and contribute to psychological distress (26). Maladaptive perfectionistic tendencies are commonly reported factors that cause high levels of stress. Burnout, with a high prevalence (7.0–86.0%) among medical students, can lead to depressive and anxiety disorders, a general decrease in well-being, and suicidal ideation. It can also affect the quality of patient care through a detached attitude and feelings of academic inadequacy. Finally, anhedonia, the inability to experience pleasure, was significantly associated with suicide attempts.

*Academic factors*: The demands inherent in medical training also play a crucial role in suicidal risk. Academic overload, excessive peer competition and perceived underperformance can generate chronic stress, exhaustion and feelings of inadequacy, increasing the likelihood of suicidal ideation (27–29). A competitive environment and high expectations are significant stressors. Burnout, directly related to academic studies, can lead to decreased performance and increased suicidal ideation.

*Psychosocial factors*: The social environment and available support are important protective factors, and their absence becomes a risk factor. Social isolation and lack of connection with others, limited family or institutional support, and difficulties in relationships with senior academics can exacerbate students’ vulnerability (30, 31). Lack of material and emotional support significantly increased the likelihood of suicidal behavior.

The stigma surrounding mental health within the medical field is a critical factor, as it can prevent students from seeking professional help for fear of being judged or discriminated against (32). Stigma related to suicide was associated with higher levels of suicide risk, poorer mental health, and lower rates of help-seeking. Medical students tend to stigmatize suicide more than other mental or physical health conditions. For socioformation, the categorization of suicide risk ceases to be a mere classification and becomes a reflective and formative process. It promotes a systemic and holistic vision in student development, oriented towards solving contextual problems through the co-creation of knowledge, collaborative work, and ethical life projects. Socioformation understands categorization as the conscious construction of psychological, academic, and social dimensions of suicide risk, not only to label patients or students, but to generate transformative learning. This means that the factors that comprise risk, such as depression, anxiety, and burnout, are analyzed in their interrelationship to build critical understanding and promote collective action. Socioformative categorization is based on complex thinking, which rejects rigid and fragmented classifications. Instead, it promotes a comprehensive understanding of the phenomenon that considers multiple factors and their interplay (33).

### 3.3 Characterization

Suicidal risk in medical students is characterized by symptoms such as hopelessness, persistent suicidal ideation, social withdrawal, decreased academic performance, anhedonia, and sleep disturbances. These indicators are not always evident, making early detection difficult (34). Often, these students do not seek help due to fear of stigma or because they feel they must demonstrate emotional strength (35).

Mental disorders represent one of the main risk factors associated with suicidal behavior, and their incidence has been steadily increasing in recent years. Epidemiological projections indicate that, by 2030, these conditions could become the leading cause of disease burden in developed countries. This projection is even more relevant considering the significant deterioration in mental health caused by the COVID-19 pandemic, which has severely impacted the psychological well-being of the population. Recent studies show that both anxiety and depression increased significantly due to imposed restrictions such as lockdowns, which, while helping to control the spread of the virus, also reduced social interaction, disrupted daily routines, and generated high economic uncertainty (36–39).

Socioformation, understood as a comprehensive educational approach (10), offers specific strategies to reduce the risk of suicidal behavior in medical students by building more humane, collaborative, and resilient learning environments. It promotes the integration of educational programs designed to develop skills such as emotional intelligence, self-regulation, and resilience, especially in high-pressure environments like medical education. Training workshops, group dynamics, and participatory methodologies, such as role-playing and peer assessment, strengthen students’ ability to cope with academic stress and emotional conflicts (40). Socioformation includes reflective activities that encourage students to develop a purposeful life project, connecting their medical vocation with values, social commitment, and existential meaning (10). This acts as a protective barrier against existential risk factors such as hopelessness, anxiety, and stress. Individualized care pathways are designed that combine early risk identification using psychometric scales with continuous clinical follow-up. The combination of self-assessments, peer mentoring, and professional assistance forms a network of ongoing support.

### 3.4 Differentiation

Suicidal ideation involves explicit thoughts, plans, or intentions to take one’s own life, while non-suicidal self-injury refers to deliberate acts of self-punishment without the intention of dying, used primarily as emotional regulation mechanisms. Although both behaviors can coexist, non-suicidal self-injury does not always predict a direct risk of suicide, although it has been shown to increase the likelihood of future suicide attempts (41). Many medical students exhibit symptoms of depression or burnout, but not all develop suicidal risk. Studies show that, although depression is a common risk factor, not all cases of depression lead to suicidal ideation; however, when symptoms of clinical depression coexist with acute stress and burnout, the likelihood of suicidal risk increases significantly (42).

Socioformation proposes instruments such as the UVE rubric (Knowing, Doing, Being/Living Together), applied to mental health phenomena. By categorizing scenarios according to low, moderate, and high-risk levels, students evaluate these cases considering not only symptoms but also values, ethical decisions, and intervention strategies (10). Implementing group dynamics where students share academic and emotional experiences fosters a culture of co-responsibility. This educational space facilitates the collective identification of early signs of suicidal risk through a process of clinical discernment that distinguishes them from other manifestations of psychological distress. Its central objective is to promote timely detection based on agreed-upon criteria, enabling essential preventive interventions. The socio-formative approach contributes to strengthening diagnostic accuracy and ethical-formative thinking, enabling students to clearly distinguish between different clinical phenomena and act with greater awareness and responsibility. Furthermore, it promotes understanding when a real risk exists and allows for the timely activation of effective intervention pathways, fostering resilience in high-stress situations.

### 3.5 Classification

The classification of suicide risk in clinical and educational contexts is based on grouping individuals according to the intensity of their ideation, the presence of previous attempts, and access to or planning of suicidal means. Generally, three main levels are defined: low risk, moderate risk, and high risk (43). The Decision Tree is a clinical tool designed to quickly and comprehensively assess suicide risk. Its main advantage is that it guides healthcare personnel with clear instructions, adapted to the individual’s risk level, facilitating precise and effective intervention. During its application, the Decision Tree is conducted as a semi-structured clinical interview, where key factors influencing suicide risk are analyzed. These include personal history such as previous attempts (chronic risk), recent signs such as agitation or hopelessness (acute risk), and access to lethal means such as firearms. Based on this assessment, the risk is classified into four levels: low, moderate, severe, and extreme (44). Socioformation proposes an educational approach that goes beyond a simple clinical classification: it seeks to transform the understanding of suicide risk from a formative, reflective, and critical perspective (10). Within this framework, the classification of suicide risk becomes a tool for developing competencies, not merely for labeling levels of severity. Instead of applying rigid risk models (low, moderate, high), socioformation promotes a classification that analyzes the phenomenon in relation to personal and academic contexts, considering individual, social, and vocational factors. This facilitates a deeper and more humane understanding of suicide risk and its use as a catalyst for transformation (45).

### 3.6 Linking

Medical students interact in demanding educational environments that foster a vulnerable emotional state. From a logical perspective, it can be postulated that academic burnout, characterized by emotional exhaustion and depersonalization, promotes states of hopelessness, which, in turn, increase the likelihood of suicidal ideation. This relationship is adequately explained by the suicide vulnerability model, where hopelessness acts as a mediating variable between emotional suffering and suicidal behavior (46) (Table 2).

**Table 2:** Logical and factual connections of suicide risk.

Construct	Logical connection	Factual evidence
Hopelessness	Pessimistic outlook, diminished sense of meaning in life causes: suicide risk	Study in Iran: students with severe hopelessness have higher levels of suicidal ideation (51)
Anxiety/depression	Chronic stress and emotional distress, and psychological deterioration causes: suicide risk	Study in China: anxiety, depression, stress, and hopelessness predict suicidal behavior (52)
Academic burnout	Emotional exhaustion and depersonalization causes: suicide risk	Research on physicians: burnout correlated with high hopelessness (46)

The table shows the relationship between suicide risk concepts based on reasoning and theory.

From the perspective of connection within the conceptual framework, the socioformative approach offers a profound way to connect knowledge with the social, ethical, and emotional context of students. This perspective allows us to understand suicide risk not only as a clinical phenomenon but also as a reflection of multiple interrelated factors: personal, academic, social, and existential. Connection through socioformation allows us to establish meaningful relationships between a phenomenon such as suicide risk and other elements of the environment. It helps connect medical students, from the educational sphere, with the emotional reality of students, as Tobón (10) emphasizes, considering emotional well-being as an essential dimension of the educational process.

Regarding suicide risk, it promotes a direct connection between educational content and the development of socioemotional competencies, such as self-awareness, emotional regulation, and ethically informed decision-making.

In the community social environment, socioformation links education with social commitment. In this case, addressing suicide risk extends to the shared responsibility among students, teachers, families, and healthcare systems, fostering a support network based on prevention, not just reaction (47). Linking the study of mental health with the development of a life plan within an ethical life project helps students find meaning and purpose in their medical training, which acts as a protective factor against suicide risk (10, 48).

### 3.7 Methodology

Several theoretical models can help understand the emergence of suicide risk in medical students. The stress-diathesis model suggests that the interaction between pre-existing vulnerability factors (diathesis, such as a family history of mental health problems or personality traits) and stressors (such as academic workload or isolation) can trigger suicidal ideation and behavior. In the interpersonal theory of suicide, Joiner (49) posits that the desire to die arises from a combination of feelings of frustrated belonging and the perception of being a burden to others, while the acquired ability to self-harm increases the risk of attempting suicide. Academic and institutional factors can contribute to these feelings of disconnection and burden.

A robust methodological approach for suicide risk prevention in medical students is the socio-formative approach, which allows for a more comprehensive and in-depth intervention. This approach articulates three fundamental components: formative projects, the appropriate use of educational technologies, and the development of an ethical life project. Through this integration, the development of metacognition is promoted, allowing students to critically reflect on their reality, strengthen their sense of purpose, and acquire tools to face personal and professional challenges with resilience. This proposal can be implemented in university educational contexts in an interdisciplinary manner, with an academic foundation and a preventive focus. A relevant feature of socioformation is fostering and building an ethical life project. For medical students, this implies a conscious and responsible orientation of their personal, academic, and professional actions, based on values such as respect, honesty, equity, and social commitment. This approach seeks not only the achievement of individual goals but also the active contribution to collective well-being and the improvement of social and health conditions in their immediate and future environment. Furthermore, it promotes a continuous search for meaning in life, which strengthens the student’s intrinsic motivation, mental health, and emotional resilience (4, 10) (Tables 3–5).

**Table 3:** Components of the training project.

Design of a training project	
Educational projects allow students to apply disciplinary knowledge to solving real problems, promoting collaborative work, self-reflection, and social commitment. In this context, we propose:	
Project theme: Promoting emotional well-being and preventing suicide in educational settings. Final product: Psychoeducational campaign for the university community (infographics, videos, social media, workshops). Assessment: Using rubrics that integrate medical knowledge, communication skills, and professional ethics.	Training projects strengthen the sense of purpose and belonging, which are protective factors against suicide risk (10, 47).

**Table 4:** Components of digital technology integration.

Integration of digital technologies	
Technology is used as a means of educational and emotional support, not just as a technical resource. It is recommended that:	
Use of digital platforms for mentoring and emotional support (e.g., support forums with teachers and peers). Design of a reflective digital portfolio where students document their emotional experiences, coping strategies, and progress in their life project. Use of validated mental health apps (such as Sanvello, MoodTools, or iPrevail) as tools for self-care and emotional monitoring.	The ethical use of technology strengthens autonomy, self-assessment, and the building of support networks (50).

**Table 5:** Components of the ethical life project.

Guided construction of the ethical life project	
The ethical life project is the central axis of prevention in social education. It is recommended that:	
Implement reflective seminars where students identify their personal, professional, and social goals. Use narrative techniques such as letters to my future self, timelines, burying a time capsule, and university life journals. Formative assessment using rubrics that consider personal commitment, ethics, decision-making, and consistency between values and actions.	Clarity of purpose in life reduces hopelessness, existential emptiness, and suicidal thoughts (4, 48).

### 3.8 Exemplification

The detailed results can be found in Table 6.

**Table 6:** Example of a socio-educational intervention campaign to prevent suicide risk among medical students.

Program title Mental health awareness campaign for doctors in training	
Project objective To design and implement an educational campaign on mental health for first- to tenth-year medical students, integrating personal experiences, scientific evidence, and social–emotional skills, with the support of digital tools and reflections on the ethical project of life.	
Socio-educational methodology	
Diagnosis	Application of validated instruments (Plutchic Suicide Risk Scale, Beck Hopelessness Scale, and Beck Depression Scale) using Microsoft Forms to assess indicators of stress, anxiety, or depression.
Ethical life project	Initial workshop: My Admission to Medical School Content: Determine whether the decision to pursue a career in medicine was based on conviction, pressure, or other interests. Technique: Write a letter to myself before participating in the workshop, place the letters in a time capsule that will be opened 3 years after graduating from medical school. Product: At the end of the workshop, present a digital self-awareness log.
Training project	Project outline: Form a team of five students, collect real testimonials while maintaining confidentiality, and support the project with bibliographic references to ensure it is scientifically sound. Upon completion, produce an academic product and share it through institutional social media channels in the form of an educational video, podcast, digital poster, or social media clip.
Use of educational technology	Open a secure channel in Teams to share resources and provide group support moderated by tutors.
Socio-educational assessment	Self-assessment with emotional analysis rubrics. Co-assessment: each team evaluates the participation of the other. Heterogeneous assessment based on relevance, academic rigor, emotional impact, and ethical commitment.
Final integrating product	ConCiencia campaign exhibition fair (digital or in-person)

## 4 Discussion

The findings of this review highlight that suicide risk among medical students is a multifactorial and dynamic phenomenon, influenced by the interaction of psychological, academic, and psychosocial factors (1, 23). The findings confirm that this population has alarming prevalences of depression, anxiety, burnout, and suicidal ideation, which are exacerbated by a demanding, competitive academic environment that often lacks institutional emotional support (2, 28). These results reinforce the need to implement early detection strategies and preventive programs in medical schools.

In terms of the academic context, it was found that work overload, competitiveness, and pressure to perform not only deteriorate mental health but also enhance feelings of hopelessness and social isolation (27, 29). This finding suggests that university environments require structural transformations, in which psycho-emotional support is an integral part of the medical curriculum.

Psychosocial factors, such as stigma toward mental health, lack of support networks, and difficulty seeking help, are established as critical barriers that increase suicide risk (32, 35). These findings coincide with those of the World Health Organization (17), which emphasizes the urgency of reducing stigma and expanding access to emotional support services.

The socio-educational approach, applied through conceptual mapping, offers a comprehensive perspective on this issue. By promoting metacognition, collaborative work, and the construction of an ethical life project, this approach favors the development of socio-emotional skills that protect against suicide risk (5, 10).

In this sense, socioformation is not limited to a pedagogical strategy but rather presents itself as a preventive and transformative model, aligned with the international call to implement sustainable and culturally relevant university mental health programs (22, 47). Thus, the discussion suggests that the approach to suicide risk must transcend the traditional clinical model to be articulated with the educational, ethical, and community dimensions, which can generate a more profound and sustained impact on medical training.

It is important to recognize the limitations of this study. In light of this, future research should consider mixed methodologies that integrate quantitative evidence with qualitative perspectives from medical students themselves, in order to enrich the contextualized understanding of the phenomenon.

One of the main strengths of this study lies in its use of socio-formative conceptual mapping as a methodological framework, which allowed for the systematic and reflective integration of the psychological, academic, psychosocial, and institutional dimensions of suicide risk among medical students. Unlike exclusively descriptive approaches, this methodology enabled not only the synthesis of available evidence but also the development of an educational intervention proposal grounded in the cultivation of cross-cutting competencies, such as metacognition, emotional self-regulation, and an ethical life project (5, 10).

In terms of future research, the results obtained open up avenues for work aimed at designing, implementing, and evaluating educational interventions based on socioformation within medical curricula. Future studies could employ mixed-methods approaches that combine the use of psychometric instruments—such as Plutchik’s Suicide Risk Scale or Beck’s Hopelessness Scale—with focus groups and in-depth interviews with students, faculty, and administrative staff, in order to capture the contextual complexity of the phenomenon (33, 47). Likewise, it is suggested to explore the role of digital technologies in suicide risk prevention through the use of virtual mentoring platforms and validated emotional self-care applications (50).

## 5 Conclusion

Medical training cannot be limited to biomedical knowledge; it is essential that educational institutions promote the overall well-being of their students, considering suicide risk as an urgent call to transform the way we educate, support, and humanize medical practice from the ground up.

Suicide risk in medical students is a complex phenomenon that needs to be understood from a comprehensive, ethical, and educational perspective. Based on the socio-educational approach and the axes of conceptual mapping, the following conclusions can be drawn:

Suicide risk is not just an individual event, but a psychosocial construct that reflects accumulated suffering, disconnection from the meaning of life, and a lack of emotional resources to cope with academic and existential demands. It manifests itself through indicators such as hopelessness, anxiety, burnout, social isolation, and deterioration in mental health, influenced by a culture of high demands, competitiveness, and little institutional support.

It can be categorized into levels (low, moderate, severe, and extreme), but prioritizing preventive actions is more important than categorization, ranging from educational support to urgent clinical intervention. Although suicide risk shares characteristics with disorders such as depression or anxiety, it is distinguished by reaching a critical clinical threshold where death emerges as a perceived solution, requiring specific prevention and monitoring strategies. Although it may seem similar to other mental disorders, such as depression or anxiety, suicide risk involves a critical threshold where the person considers death as a way out, which requires specific prevention and follow-up strategies. Suicide risk is connected to key constructs such as hopelessness, anxiety, burnout, and the absence of an ethical life project. These connections allow for the design of educational and training interventions that not only detect but also prevent emotional deterioration.

This proposal addressed the elements of socio-formative assessment: prior knowledge, expected outcomes, observable performance, co-assessment, metacognition, and transfer, with the intention of training future physicians not only in science but also in humanity and resilience.

## Data Availability

The original contributions presented in the study are included in the article/supplementary material, further inquiries can be directed to the corresponding author.
